# Multi-agent approach to sequence structure simulation in the RNA World hypothesis

**DOI:** 10.1371/journal.pone.0238253

**Published:** 2020-08-28

**Authors:** Jaroslaw Synak, Agnieszka Rybarczyk, Jacek Blazewicz

**Affiliations:** 1 Institute of Computing Science, Poznan University of Technology, Poznan, Poland; 2 Institute of Bioorganic Chemistry, Polish Academy of Sciences, Poznan, Poland; 3 European Center for Bioinformatics and Genomics, Poznan, Poland; "INSERM", FRANCE

## Abstract

The origins of life on Earth have been the subject of inquiry since the early days of philosophical thought and are still intensively investigated by the researchers around the world. One of the theories explaining the life emergence, that gained the most attention recently is the RNA World hypothesis, which assumes that life on Earth was sparked by replicating RNA chains. Since wet lab analysis is time-consuming, many mathematical and computational approaches have been proposed that try to explain the origins of life. Recently proposed one, based on the work by Takeuchi and Hogeweg, addresses the problem of interplay between RNA replicases and RNA parasitic species, which is crucial for understanding the first steps of prebiotic evolution. In this paper, the aforementioned model has been extended and modified by introducing RNA sequence (structure) information and mutation rate close to real one. It allowed to observe the simple evolution mechanisms, which could have led to the more complicated systems and eventually, to the formation of the first cells. The main goal of this study was to determine the conditions that allowed the spontaneous emergence and evolution of the prebiotic replicases equipped with simple functional domains within a large population. Here we show that polymerase ribozymes could have appeared randomly and then quickly started to copy themselves in order for the system to reach equilibrium. It has been shown that evolutionary selection works even in the simplest systems.

## Introduction

According to the RNA World theory, early evolution of life involved RNA molecules both as genetic information carrier and catalyst (enzyme) [1]. Strong support for this hypothesis came from the discovery of the self-splicing RNAs [2], the RNase P [3] and small endonucleolytic ribozymes such as hairpin, hammerhead and HDV [4–6], which are capable of cleaving RNA molecules. Additional evidence for RNA world preceding modern life includes the informational and catalytic role of RNA in translation, splicing and gene expression [7]. These findings, along with ongoing RNA-oriented research suggest that the synthesis and replication of RNA were the critical processes in the transition from prebiotic chemistry to life.

One of the crucial questions in the origin of life is how replicating functional molecules could have appeared, survived and evolved towards more complex systems. A partial answer is offered by the template-directed self-replication of RNA polymerase ribozyme (replicase) [8]. Since the ancestral replicase has been entirely replaced by a peptide replicase, to resolve whether self-replication of RNA is possible, researchers have devoted a considerable effort to construct through artificial evolution, ribozymes that are able to synthesize RNA oligonucleotides in a template-dependent manner [7, 9–14]. Among different existing ribozymes obtained by *in vitro* selection, 24-3 polymerase ribozyme is the most proficient one [15] and recently isolated ribozyme accepting 5’-triphosphorylated RNA trinucleotides (triplets) as substrates is able to conduct RNA-catalysed RNA synthesis on even highly stable and complex RNA structures, which previously was the significant obstacle that in most cases hindered the RNA replication [16]. Ribozymes like those mentioned above, are very promising, but researchers are still far from the discovery of the ancient ribozyme with intact replication activity and many challenges will have to be overcome to show that RNA itself could have supported evolving genetic system [17].

Another significant problem related to the origins of life concerns lack of the error-correction mechanisms and frequent mutations that must have been faced by the prebiotic replicator sets [18]. Hence, as a consequence, evolution forms a population of replicons (quasi-species) being a collection of RNA replicases and interrelated cloud of mutants (parasites defined as RNA molecules that do not possess replicase activity, but can be replicated by RNA replicases). In such system, the length of replicases is restricted by the accuracy of the replication, which is error-prone due to mutations. Furthermore, if the mutation rate increases above some threshold value, an error catastrophe (which is a complete loss of information) occurs. Based on the highly simplified model, Maynard Smith [19] estimated the length of the polynucleotide chain which, while preserved, guarantees that the information can be sustained in the quasi-species. According to his analysis, for a given error rate per base replication, the upper limit of genome size, in the absence of enzymes, is around 100 nucleotides [19]. This observation is defined as Eigen paradox, where in order to be a functional replicator, RNA molecule must be long enough, but being such, it cannot be maintained in the population since it will be quickly overtaken by parasites [18, 20–26]. It constitutes the fundamental obstacle to increase in complexity and is subsequently summarized as: no enzymes without a large genome, and no large genome without enzymes [18, 27–29]. As a solution to the problem mentioned above, Eigen suggested hypercycles [21], where a number of molecules catalyse the replication of each other in a cyclic way, what allows to combine their information and cross the error threshold [22, 24]. However, Maynard Smith [19] raised important objection that hypercycles are vulnerable to parasites in homogeneous solutions [22, 25, 30–33]. It has been shown, that while such system is able to maintain its diversity, it is evolutionary and ecologically unstable and its evolvability is limited [34, 35]. Boerlijst and Hogeweg were the first to show that the problem of negative impact of parasites can be reduced by considering spatially organized systems [22]. Moreover, Hogeweg together with Takeuchi proposed an alternative formalism to model hypercycle dynamics with spatial extension, so-called stochastic cellular automata (CA), characterized by the locality of interactions and the discreteness of a population [31–33]. They showed that replicase-parasite system (*RP model*) is resistant against parasites and that parasites form traveling wave patterns being the consequence of spatial self-organization and resulting in evolutionary stability of the system [31, 33].

Additionally, Takeuchi and Hogeweg investigated also the evolution of the complexity in a simple RNA-like replicator system by applying Monte Carlo simulation [36]. They introduced RNA sequences and their secondary structures (RNA 2D structures, which are in general represented by a list of the nucleotide bases paired by hydrogen bonding within its nucleotide sequence) into the model. The replication process was dependent here directly on the arbitrarily defined structure and base-pairing matching among dangling-ends of interacting molecules. The simulation based analysis conducted by them, showed that their system could survive and produce several coexisting species.

The above mentioned replicase-parasite surface model developed by Takeuchi and Hogeweg was deepened and refined in [25], where more general approach based on multi-agent systems (MAS) with more realistic assumptions regarding the movement of entities (diffusion) has been proposed. MAS consists of a set of agents capable of making autonomous decisions and interacting in a given dynamic environment. When compared to CA, MAS approach is more suitable for simulating biological systems since it provides easier way for representing interactions between replicons through agent intercommunication. During the simulations conducted by the authors, mesoscopic entities resembling traveling waves were observed, but because of the more continuous treatment of the space, they looked more like explosions of life [25].

The RP system modelled and implemented using multi-agent modelling technique and described above, constitutes still a great simplification of a real prebiotic set. Each agent (replicase or parasite) is equipped with only two parameters, namely the affinity towards replicases (models how well the molecule is recognized by the replicase and serves as a replication template) and the probability of being in the folded state. In our work, in order to introduce more realistic biological assumptions, the MAS approach introduced above has been extended to take into account the RNA sequence and structure. The model and simulation algorithm have been modified by adding RNA sequence information to every agent, and what follows, the agent’s parameters are now directly derived from its exact sequence. Here, contrary to [36], it is assumed that only RNA containing characteristic motif (domain) is able to act as an RNA polymerase ribozyme (replicase). Since the motif of that kind has not yet been discovered and the most important aspect in the evolutionary dynamics is the correlation between a sequence and its function rather than the real sequence itself, we decided to use arbitrarily presumed motif coming from the engineered and evolved in the laboratory polymerase ribozyme [7]. Additionally, unlike [36], the mutations together with primary and secondary structure of the aforementioned motif influence the RNA replicase efficiency, which is common in modern life. By introducing mutation rate which is very similar to the real one, it became also possible to observe very simple evolutionary mechanisms, which could have led to the more complicated systems and eventually, to the formation of the first modern cell. The main goal of this study was to determine the conditions that allowed the emergence and evolution of the prebiotic replicases containing simple functional domains within a large population and to analyze their influence on the survivability of the system. Here we show that polymerase ribozymes could have appeared randomly and then quickly started to copy themselves in order for the system to reach equilibrium. This shows that evolutionary selection works even in the simplest systems. Parasite and replicase populations have the ability to regulate each other and their mutual interaction can result in equilibrium.

## Materials and methods

### Replicase-Parasite model (RP model)

RP model used and extended in this article was presented in [32], but there are also additional assumptions which were described in [25] and are taken into account. The population being simulated consists of RNA molecules, where every RNA molecule can diffuse freely, but also has a limited lifetime after which it decays. This reflects the real behaviour of the chemical molecules. Every RNA molecule has two parameters associated with it:

- *l* - fraction of time spent in the folded state. It is assumed that folding and unfolding occurs so quickly that there is no need to represent it explicitly in the simulation and the molecule state is determined randomly whenever necessary.
- *a* - affinity towards replicases, the greater it is, the easier for the agent is to be copied

RNA strands are divided into replicases and parasites. Replicases can act as enzymes and catalyze replication of themselves and of other molecules (they have also an additional parameter *a*—efficiency of replication), whereas parasites can be copied by replicases, but cannot catalyze replication themselves. Before replication can occur, a complex must be formed. Complexes consist of two RNA molecules, where one serves as an enzyme (replicase) and second serves as a template (replicase or parasite). One of the most important assumptions is that a complex stabilizes both its parts, so they cannot decay in the simulation (i.e. complex has to dissociate before that happens). As soon as the complex is created, it starts to perform replication and dissociates after it is finished. During replication, the template is copied, but the process is not perfect, so the newly created strand can have slightly different values of its parameters (random mutations).

A complex can be formed when two RNA molecules are close enough to each other (closer than the interaction radius) and at least one of them is a replicase. Complex formation is a stochastic event, its probability can be computed using parameters of the template:

$$\begin{array}{c} p_{\text{formation}}=a\left(1-l\right) \end{array}$$ (1)

It represents the fact that during complex formation, the template has to be unfolded to be accessible for the enzyme (replicase).

### Extended Replicase-Parasite model (extended RP model)

In this section, our approach, which is an extension of the RP model has been described. In order to build a model that is closer to reality, some modifications presented below, had to be made.

#### General assumptions

The main difference in relation to the approach presented in [25], is of course the explicit introduction of RNA sequences. Previously agent’s parameters were arbitrary, now they are derived from its sequence. During agent’s mutation its parameters aren’t modified directly anymore, rather the agent’s sequence changes and thus its parameters. In order to build a model that is closer to reality, we used the information of the primary and secondary RNA structure, hydrolysis rate of the phosphodiester bonds and we have introduced the characteristic catalytic motif within RNA replicase. Certainly, our goal was not to discover and explicitly identify the motif content (length, sequence etc.), but rather to observe the evolution dynamics.

Besides this main difference, three minor changes had to be made in the algorithm in order to ensure that the parallel execution would be effective:

- complexes cannot diffuse
- complexes cannot dissociate before replication
- parameter *a* now denotes the efficiency of the replicase, for parasites this parameter is equal to 0

**Fig 1:**
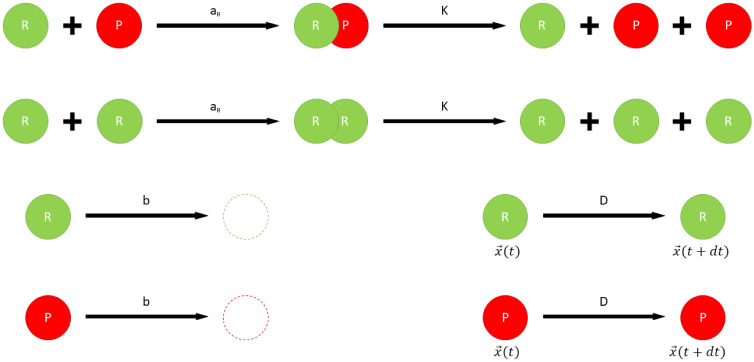
All interactions allowed in the simulation. Replicase (R) can form a complex with other replicase (R) or with parasite (P) at *a*_*R*_ rate. A replicase-replicase (RR) or replicase-parasite (RP) complex can produce new entity (replicase in a case of RR and a parasite in the situation of RP) at rate *K*. Parasites and replicases can move at rate *D* and decay at rate *b*. Vector $\vec{x}\left(t\right)$ denotes the agent’s position and vector $\vec{x}\left(t+dt\right)$ denotes its position in the next step.

Fig 1 shows all molecular interactions (attractive or repulsive forces between molecules) possible in the simulation.

#### Hydrolysis rate of the phosphodiester bond

The most detailed study of the molecular mechanisms that underlie the process of RNA structure-driven spontaneous RNA hydrolysis in the absence of any protein enzymes (i.e. non-enzymatic RNA degradation/decay) was conducted by Kierzek and co-workers [37–40]. They analysed the chemical stability of individual phosphodiester bonds in the following conditions. The circa 0.1 pmol of ^32^P 5’-phosphorylated oligoribonucleotides were incubated in the reaction mixture consisting of: 1 mM EDTA, 1 mM spermidine, 50 mM NaCl, 0.1% PVP (polyvinylpyrrolydone) and 50 mM Tris-HCl (pH 7.5) at 37°C. Next, the reaction products were separated by electrophoresis on 20% polyacrylamide gels and visualized by autoradiography [37–40]. It is worth noting that the conditions mentioned above are similar to those that are present in the eukaryotic cells, which are certainly different from the prebiotic ones. Unfortunately, to our knowledge, Kierzek and co-workers are the only ones who quantitatively analyzed the reactivity of the phosphodiester bonds within RNA molecules and developed the rules displaying their stability.

As a result, they reported, that distinct phosphodiester bonds are cleaved with different rates and that the cleavage occurs preferentially within single-stranded regions of the RNA molecule. They showed, that YA (Y being pyrimidine) bonds were 3-5 fold more sensitive to hydrolysis than YC. Additionally, CA was 1.5-2.0 fold more stable than UA. The phosphodiester bonds YA and YC were 20 fold more susceptible to cleavage than YG and YU, while RR and RY (R being purine) were stable under the applied conditions. They also noticed, that the pattern of the non-enzymatic hydrolysis of RNA molecules was similar to that of ribonuclease A (RNase A), which could reflect an evolutionary process. Hence, if RNA was originally hydrolysed at the sites that were susceptible to cleavage, then it is likely that the same cleavage sites would be favoured by the proteins, that evolved later [40].

The hydrolysis rates of the phosphodiester bonds in the simulation were calculated basing on the aforementioned results obtained by Kierzek [37–40] and those presented in [41].

#### RNA sequences

Every agent has an RNA sequence with a length of 50 nucleotides associated with it. It is worth noting that naturally occurring ribozymes vary in size with the smallest one being the hammerhead (40 nucleotides in length) [42]. Additionally, currently engineered and evolved in the laboratory putative replicases are long (around 200 nucleotides) and what follows, unlikely to be discovered randomly [43]. Moreover, it has been suggested that evolution proceeded in stages, thus shorter functional molecules could precede complex replicases. It has been also shown in [43], that a common feature of many essential ribozymes including putative polymerase ribozymes is multibranched secondary structure, which can already be found in the RNA molecules with sizes above 40 nt. Additionally, the length of 50 nt appears to be the upper limit for non-enzymatic self-replicating processes [43, 44].

Based on this sequence, agent’s parameters are computed. Currently, all sequences have the same length (one of the simulation parameters) and set of nucleotides identical with the real RNA world (A, C, G and U), although it is not theoretically necessary since any set of symbols can be used. When a sequence is processed, first its folded form (secondary structure) is determined (by properly pairing nucleotides). Secondary structures are predicted using IPknot, a tool with Vienna RNA Package as its base, capable of predicting pseudoknots [45].

#### Sequence motif of RNA replicase

Specific, conserved and functional motifs are widespread among the genomes of modern organisms. As far as RNA is concerned, they can have an unstructured (e.g. motifs documented in non-coding RNAs, responsible for mRNA processing, translation and degradation [46]) as well as structured form (e.g. conserved secondary structure patterns and consensus sequences in the catalytic core of hammerhead ribozymes [47]). Unlike RNA, protein motifs usually have structural context. For example, in a case of all RNA-dependent RNA polymerases (RdRp), which are very ancient enzymes of RNA viruses, responsible for carrying out replication and transcription of their genomes, several conserved structural motifs (divergent in their sequences) have been identified in the proximity of the catalytic site, indicating their functional importance in enzymatic activity [48, 49].

Here, it is assumed that only RNA containing characteristic motif (domain) located at its 5’-end [50] is able to act as an RNA polymerase ribozyme (replicase), which is consistent with the previous studies [7, 50]. Unfortunately, the motif of that kind has not yet been discovered. Additionally, self-replicating systems of RNA molecules have not been found in nature, but scientists have made significant progress toward constructing them through biochemical experiments [7, 9, 10, 16, 51]. Furthermore, it has been shown in laboratory, that a short single-stranded sequence segment located at 5’ end of the replicase significantly influences its activity [7]. It is the reason why, we decided to use arbitrarily presumed motif, consisting of twenty nucleotides derived from 5’-end of the evolved and engineered polymerase ribozyme tC19Z [7], namely *UCAUUGAAAAAAAAAGACAA*.

It should be noted, that the most important aspect in the evolutionary dynamics is the correlation between a sequence and its function rather than the real sequence. In general, any other motif could have been taken into account, similarly as the structure and sequence of the prebiotic self-replicting ribozymes could have been and probably were quite different from those obtained in the laboratory. The main goal of this study was to determine the conditions that allowed the emergence and evolution of the first replicases containing functional domains and show that it was potentially possible.

### Description of the simulation algorithm

The algorithm is based on the approach described in the previous section. The execution is parallelized in order to shorten the computation time.

#### Parameters

In this subsection, the parameters used in the simulation are described, both global (see Table 1) and individual for each RNA strand.

**Table 1:** Parameters used in the simulation. They are global constants that are used extensively in the algorithm.

Parameter name	Value	Description
*int*_*radius*	3	Radius of a single agent
*sizeX*, *sizeY*	1000	Simulation area size
*seq*_*length*	50	Agent’s sequence length, constant for all agents
*d*	0.01	Base decay rate
*seq*_*mut*	0.01	Mutation probability of a single nucleotide
*dt*	0.1	Single step length (Δ*t* in equations)
*D*	4	Diffusion constant
*K*	∞	Replication rate
*selProb*	0.01	When the simulation is finished, random agents are selected and their sequences saved to the resulting file. For every agent the decision is made randomly (and independently) - it can be chosen with probability *selProb*.
*initR*	Depends on the scenario	Initial number of replicases (agents with a predefined replicase sequence)
*initP*	Depends on the scenario	Initial number of parasites (agents with random sequence, some of them can randomly be replicases)
*neigh*	4	Maximum number of neighbours for an agent
*d*(*NN*)	0	Hydrolysis rate of the phosphodiester bond connecting neighbouring nucleotides in the RNA strand. *N* corresponds to any base, meaning: A, C, G or U.

#### Agent’s parameters

Every agent has three parameters associated with it:

- *l* - fraction of time spent by RNA molecule in the folded state (unable to be copied),
- *a* - replicase efficiency (if it is equal to 0 then the agent is a parasite),
- *b* - decay rate,

Based on these three parameters, agent’s behaviour is determined.

*Fraction of time spent in the folded state* (*l*). The value of this parameter is equal to the number of paired nucleotides divided by the total sequence length.

*Replicase efficiency* (*a*). The computation of the replicase efficiency consists of two stages. First, the replication rate *k*_*R*_ is computed (it can be any number greater than or equal to 0). It should be noted that during these computations only the beginning of the sequence (first 20 nucleotides) is considered. The result rate is equal to the number of nucleotides identical with the replicase template (*UCAUUGAAAAAAAAAGACAA*), multiplied by 10. However, if the number of those nucleotides is less than 7, the rate is set to 0 (agent is a parasite). In the second stage, the replication probability (during the current step) is computed (agent’s parameter *a*) using the equation:

$$\begin{array}{c} a=1-e^{-k_{R}\Delta t} \end{array}$$ (2)

This equation was derived from the assumption that the probability of reaction is equal in every moment, which means that Poisson distribution [52] can be applied here. The equation above represents the probability that after time Δ*t*, the reaction occurs.

*Decay rate* (*b*). It is assumed that decay can occur spontaneously. Agent’s decay rate is calculated as a sum of base decay rate (simulation parameter *d*—see Table 1) and hydrolysis rates of the phosphodiester bonds assigned to every pair of neighbouring nucleotides (also given as simulation parameters in Table 1). For example, in case of RNA sequence UUUACG, four hydrolysis rates will be added: *d*(*UU*) (twice), *d*(*UA*), *d*(*AC*) and *d*(*CG*).

It is worth noting that apart from the dissociation, decay is the only unimolecular reaction in the system (see “Unimolecular reactions” subsection for details).

#### Mutation

Only substitutions are considered. During mutation, every nucleotide has a chance (simulation parameter *seq*_*mut*) of being replaced by another (randomly chosen, but the new nucleotide cannot be the same). Each nucleotide can mutate independently.

#### Ideal motif-driven RNA replicase

In the beginning of the simulation, two types of agents are put in the simulation area: agents with completely random sequences (parameter *initP*) and ideal replicases (parameter *initR*). Ideal replicase is an agent with the best possible efficiency, having first 20 nucleotides equal to *UCAUUGAAAAAAAAAGACAA*. Next twenty nucleotides within agent’s sequence are equal to *UUGUCUUUUUUUUUCAAUGA*, which is the complementary sequence to the first fragment, the last ten nucleotides are chosen randomly. The average *l* parameter of such replicases is 0.76.

#### Unimolecular reactions

The approach to the unimolecular reactions is similar as in the previous algorithm [25]. Instead of checking during every step whether the reaction had occurred, algorithm in the beginning computes how much time has to pass for the reaction to happen and during every step, this time is decreased.

$$\begin{array}{c} t_{action}=-\frac{ln(X)}{k} \end{array}$$ (3)

where *t*_*action*_ is the time that has to pass for the reaction to start, *X* is a random variable distributed uniformly in the range (0;1] and *k* is an average reaction rate. Time computed basing on the above equation is expressed in time units (depends on the unit of *k*). However, in order to be used in the simulation, it has to be expressed as the number of simulation steps:

$$\begin{array}{c} {t_{action}}^{\prime }=\left\lfloor \frac{t_{action}}{\Delta t}\right\rfloor +1 \end{array}$$ (4)

In this article, this approach is used only for the decay, as dissociation happens always right after replication.

#### Diffusion

Brownian dynamics is used for diffusion in the simulation. Only single agents can diffuse, while complexes are assumed to be “heavier”, thus their diffusion is not explicitly performed. During diffusion, each agent is moved by a random vector, computed according to the following formula:

$$\begin{array}{c} \vec{w}=\sqrt{6\Delta tD}p\vec{\epsilon } \end{array}$$ (5)

where *p* is an evenly distributed random number from the range [0;1], *D* is the diffusion constant and $\vec{\epsilon }$ is a random unit vector. It can be proven, using central limit theorem that after sufficiently many steps, this approach gives very similar results to the traditional one [53]:

$$\begin{array}{c} \vec{w^{\prime }}=\sqrt{2\Delta tD}\vec{\epsilon^{\prime }} \end{array}$$ (6)

where $\vec{\epsilon^{\prime }}$ is random Gaussian vector with unit variance.

#### Complex formation

Agents which are closer to each other than interaction radius (simulation parameter *int*_*radius*—see Table 1) can form a complex. When two agents form a complex, all their parameters are memorized (including remaining lifetimes) and two unimolecular reaction times are computed: time to dissociation and time to replication. We assume that forming a complex stabilises both RNA strands, so agents involved, do not “age” until the complex dissociates. Complexes also do not diffuse.

Agent which initiated the complex formation is considered a replicase (subscript *E*) and it has to be in the folded state in order to be active (real life enzymes are functional, because of their unique shape). The second agent serves as a template (subscript *T*) and will be copied during replication, it can be either a parasite or a replicase. In order to step into the reaction, it has to be unfolded to ensure that the whole sequence is accessible during replication. Considering this, the probability of complex formation, when two agents meet, can be computed from the following formula:

$$\begin{array}{c} reaction\_probability=a_{E}*l_{E}*\left(1-l_{T}\right) \end{array}$$ (7)

where *a*_*E*_ and *l*_*E*_ are first agent’s (replicase) replication efficiency and its probability of being in the folded state, *l*_*T*_ is the probability of being in the folded state for the second agent (template).

#### Replication

The replication procedure is as follows. The template is copied and new agent (with the same sequence) is put in the same position. Next, the mutation is performed, so the resultant sequence can be slightly different from it’s template. After replication, the complex dissociates.

#### Dissociation

During dissociation both agents are “released”. They can move freely and their “aging” is resumed.

### Implementation details

The algorithm was implemented using C++ programming language and OpenMPI package.

The whole algorithm is presented below in the form of the pseudocode. Some purely implementational details have been omitted for simplicity. The code is executed by each thread separately. The command “synchronize” waits for all threads to execute and then the program execution can continue. The *rank* variable denotes the current thread id, whereas *size* is the total number of threads.

**Algorithm 1**: Main loop

**1** **for** *step* ← 1 **to** *max_steps* **do**

**2**  Diffusion and decay (Algorithm 2);

**3**  **synchronize**;

**4**  Simulate interactions (Algorithm 3);

**5**  **synchronize**;

**6** **end**

**Algorithm 2**: Diffusion and decay

**1** Make list *list* of thread’s agents ordered randomly;

**2** **while** *list not empty* **do**

**3**  Take first agent *A* from *list*;

**4**  **if** *A is complexed* **then**

**5**   Decrease *A* replication time;

**6**   **if** *A replication time is* **0 then**

**7**    Replicate *A*’s complex and perform mutation for newly created agent;

**8**    Dissociate *A*’s complex;

**9**   **end**

**10**  **end**

**11**  **else**

**12**   Decrease *A* lifetime;

**13**   **if** *A lifetime is* **0 then**

**14**    Remove agent *A* from simulation;

**15**   **end**

**16**   **else**

**17**    Diffusion for agent *A*

**18**   **end**

**19**  **end**

**20** **end**

**Algorithm 3**: Interactions of agents

**1** Make list *list* of thread’s agents ordered randomly;

**2** **while** *list not empty* **do**

**3**  Take first agent *A* from *list;*

**4**  **if** *A is not complexed* **then**

**5**   Make list *list*2 of agent’s neighbours ordered randomly;

**6**   **if** *Size of list*2 *is greater than simulation parameter neigh* **then**

**7**    Remove agent *A* from simulation;

**8**   **end**

**9**   **else**

**10**    **while** *list*2 *not empty* **do**

**11**     Take first agent *n* from *list*2;

**12**     **if** *n is not complexed* **then**

**13**      *reaction_probability* = *A*.*a* * *A*.*l* * (1 − *n*.*l*);

**14**      Take a uniformly distributed random number *p* from [0; 1);

**15**      **if** *p* < *reaction_probability* **then**

**16**       Form complex of *A* and *n*;

**17**      **end**

**18**     **end**

**19**    **end**

**20**   **end**

**21**  **end**

**22** **end**

## Results

The main goal of the simulation was to analyze the behaviour of the large population of RNA molecules. Using multi-agent system described in the previous section, we conducted several simulations to check different conditions. We analyzed the case when the initial population consisted only of ideal replicases and the case when in the beginning of the simulation all RNA sequences were completely random and some of them happened to have accidentally proper replicase sequence. We also considered the scenario in which we checked how the additional information concerning the chemical stability of individual phosphodiester bonds connecting neighbouring nucleotides in the RNA strand influences the simulated population. Each time, the parameter values *a*, *l* and the changes in the simulated population size are presented on the charts. These values correspond to the average values taken from the whole simulated population in each simulation step.

The analyzed system is considered alive if there is at least one RNA strand in the simulation area. Simulations were conducted until the system went extinct (there was no RNA strands anymore) or reached stability (nothing was changing over long time).

### Parameters values used in the simulations

Parameters used in simulations (see Table 1) were mostly chosen empirically. The main goal was to have large population, which is still possible to simulate in reasonable time. Base decay rate and mutation rate values were chosen to accelerate system evolution (in order to observe evolutionary trends in reasonable time) without driving it to extinction. Hydrolysis rates in Scenario 3 were based on real life values, measured for non-enzymatic decay of RNA [37–40].

### Scenario 1

**Fig 2:**
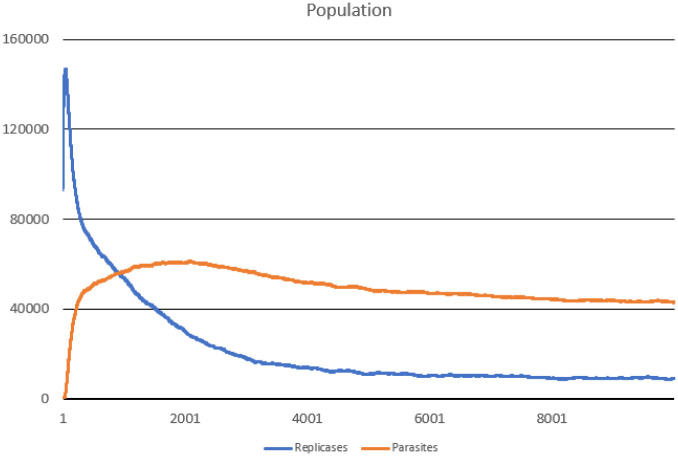
Population of parasites and replicases in Scenario 1.

**Fig 3:**
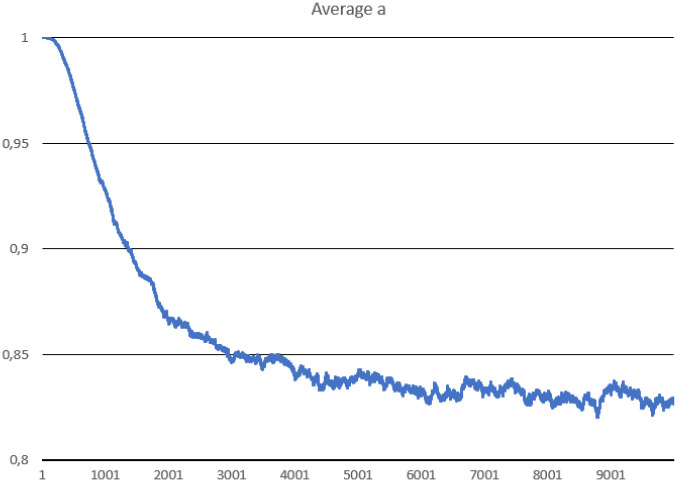
The average replication efficiency of replicases in Scenario 1.

**Fig 4:**
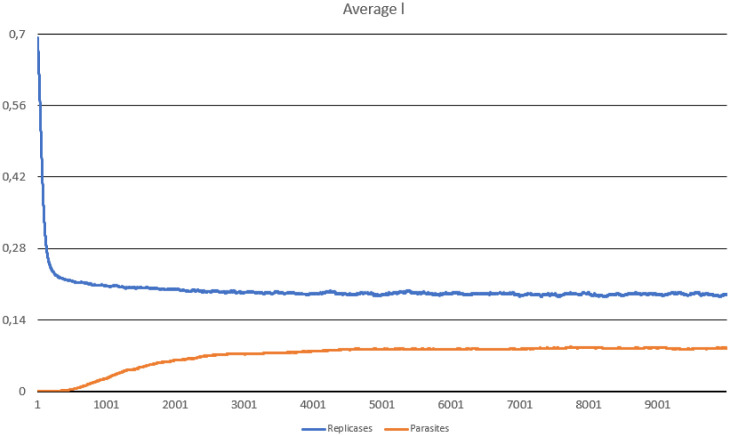
The average probability of being in the folded state, computed for parasites and replicases in Scenario 1.

In this scenario, one hundred thousand ideal replicases were randomly placed in the simulation area. The goal was to check the stability of population consisting of replicases only and observe if parasites can appear and drive the whole system to extinction. During the simulation, multiple important values were measured, such as: the number of replicases and parasites, average replication efficiency and average *l* values for replicases and parasites (separately). The number of simulated steps was 10000, but after 8000 steps the system reached stability. Results are presented in Figs 2, 3 and 4.

The system survives without any problem and all parameters stabilize after a period of time. In the beginning, replicases rapidly increase in number, but this trend is reversed by emerging parasites (some sequences were copied with errors and as a result, they lost the ability to replicate RNA). The replicase population shrinks significantly, in the same time parasties reach their peak and their number also dwindles a little bit (Fig 2). At the end of the simulation, both populations stabilize. Very interesting thing is that *l* parameter is on average much lower for parasites (Fig 4), which means that they are much easier to copy than replicases. Despite of this, the population of parasites is “kept in check” by the system. Last, but not least, the average *a* parameter starts from the optimal value in the beginning, but slightly decreases over time due to mutations and eventually becomes stable (Fig 3).

### Scenario 2

In this scenario, all RNA sequences were completely random. Some of them by accident had a proper replicase motif. This situation is much closer to the one, where first RNA molecules appeared randomly. Certainly, whether random sequences can kick-start evolution is an unsolved question. However, the results presented here show that if some replicases emerge from a random pool of RNA sequences, then their population can grow and take over the system, which can finally reach stability.

**Fig 5:**
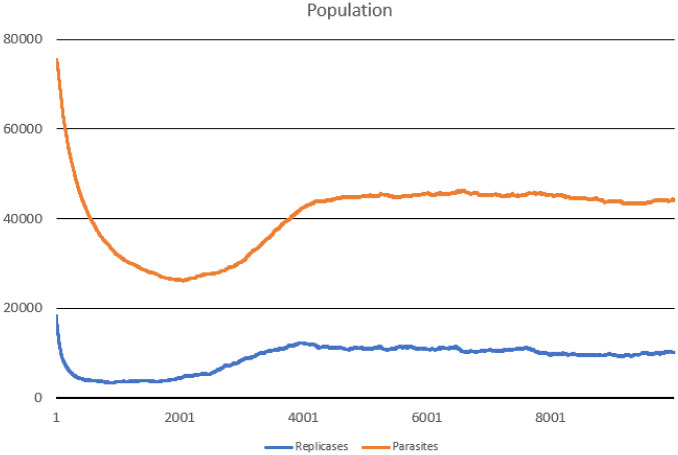
Population of parasites and replicases in Scenario 2.

**Fig 6:**
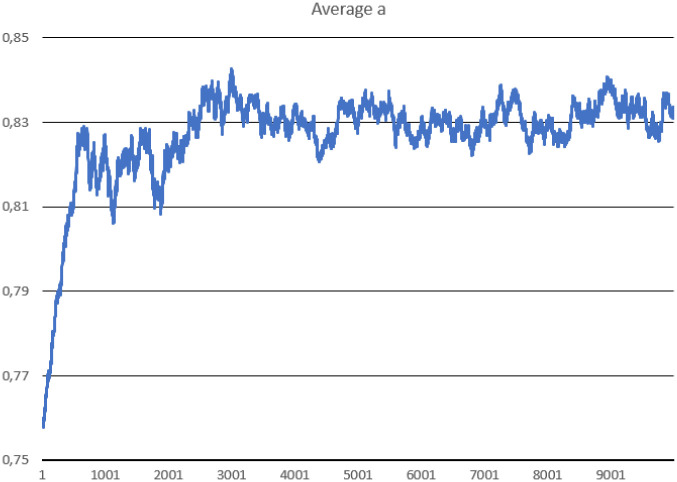
The average replication efficiency of replicases in Scenario 2.

**Fig 7:**
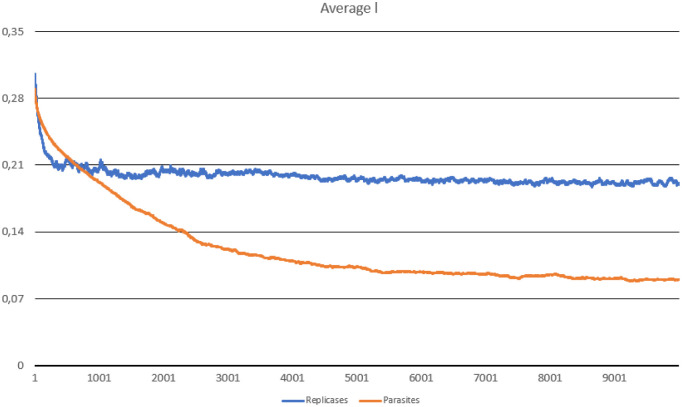
The average probability of being in the folded state, computed for parasites and replicases in Scenario 2.

The number of steps simulated was the same as in the previous scenario 10000. Results were presented in Figs 5, 6 and 7.

Evolution of the system is very interesting. First, most of the parasites decayed, because they did not have any access to replicases. Population of replicases started to diminish as well, because the majority of them was too weak to sustain the tiny population (Fig 5). As a result, the average replication efficiency (Fig 6) started to increase very sharply (natural selection). Replication became much more effective, so the population of replicases started to grow. When the population of replicases increased, the population of parasites started to increase as well (they started to be copied by replicases), eventually both populations became stabilized.

The last interesting thing was the evolution of average value of *l* parameter (Fig 7), as it started to decrease for both parasites and replicases until it reached stability.

### Scenario 3

**Fig 8:**
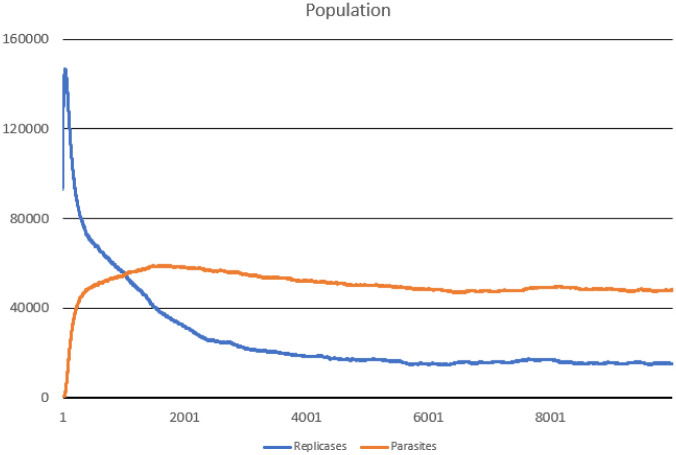
Population of parasites and replicases in Scenario 3.

**Fig 9:**
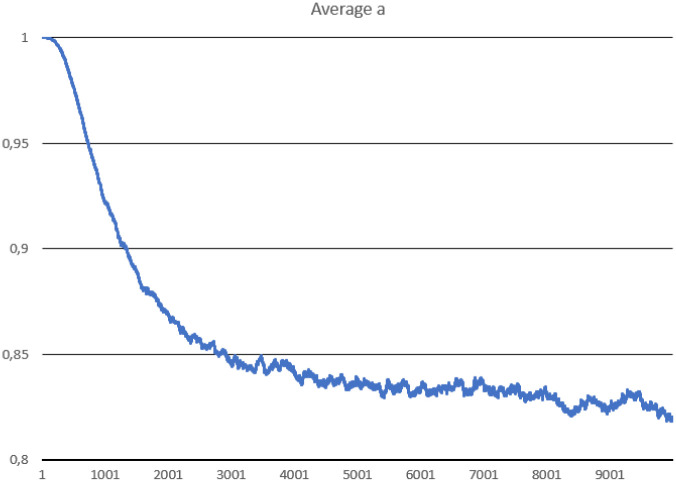
The average replication efficiency of replicases in Scenario 3.

**Fig 10:**
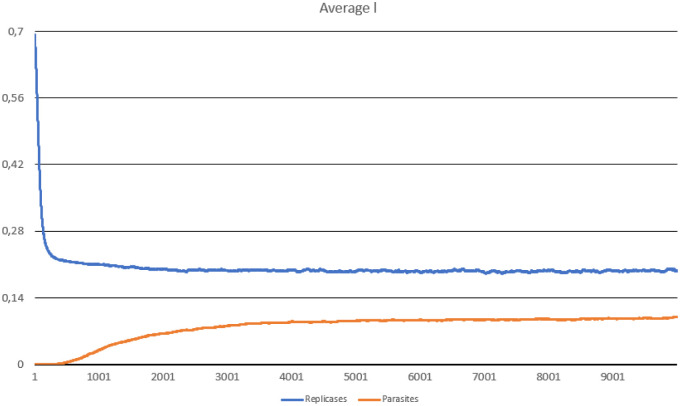
The average probability of being in the folded state, computed for parasites and replicases in Scenario 3.

This simulation was carried out to check the influence of *d*(*NN*) parameters on the simulated population. Hydrolysis rates of the phosphodiester bonds are presented in Table 2. All hydrolysis rates not shown in the Table 2 are equal to 0. In the beginning 100000 replicases were placed in the simulation area, replicase efficiency was computed exactly like in the Scenario 2. Despite higher decay rates (because of the hydrolysis of the phosphodiester bonds), the simulated population managed to survive (results are presented in Figs 8, 9 and 10).

**Table 2:** Hydrolysis rates of the phosphodiester bonds (*dNN*) used in the Scenario 3.

Phosphodiester bonds	Hydrolysis rate of the phosphodiester bonds *d*(*NN*)
UA	0.000953
CA	0.000932
UC	0.000846
CC	0.000846
UG	0.000100
CG	0.000100
UU	0.000100
CU	0.000100

It is worth noting that cytosine and uracil were partially eliminated from the system (occurred much less frequently in the sequences). Despite of this, the results are almost the same as in the Scenario 1.

### Scenario 4

In this scenario, simulations were carried out taking into account different values of parameters in order to check how they influence the results and the simulated population was smaller as compared to previous scenarios. The main goal was to show the parameters ranges where the system collapses versus survives. Two parameters were tested:

- base decay rate (*d*) - its value determines how long a single RNA molecule can live or how often one generation has to be replaced by the next one. This has a great influence on the speed of the evolution.
- diffusion constant (*D*) - this parameter determines how important is the spacial aspect. The lower *D* is, the less homogenous the system becomes, because the diffusion is slower.

The results are presented in the Tables 3 and 4.

**Table 3:** System parameters values after 10000 steps of simulation in case of different values of base decay rate *d* (Scenario 4).

Base decay rate *d*	Population size	Average *a* (replicases)	Average *l* (replicases)	Average *l* (parasites)
0.001	390	0.83	0.19	0.09
0.01	680	0.82	0.17	0.12
0.6	167	0.86	0.26	0.13
0.7	0	n/a	n/a	n/a

**Table 4:** System parameters values after 10000 steps of simulation in case of different values of diffusion constant *D* (Scenario 4).

Diffusion constant *D*	Population	Average *a* (replicases)	Average *l* (replicases)	Average *l* (parasites)
0	0	n/a	n/a	n/a
4	680	0.82	0.17	0.12
40	351	0.65	0.15	0.08
50	651	0.77	0.16	0.08
100	616	0.79	0.17	0.07
400	619	0.81	0.18	0.08

The results show that for lower values of base decay rate *d*, the evolution of the system is slower and also the population becomes smaller, but that is the only significant difference. For greater values of this parameter, RNA molecules have no chance to evolve and can barely survive or they die out completely. In the situation of diffusion constant *D*, if it is slightly faster, then the system survives, but with smaller population. In case of significantly bigger values of *D*, there is no difference besides growing *a*. However, slower diffusion has a big impact on the system, as replicases can’t spread effectively and eventually the population dies as a result.

## Discussion

In this paper, we have described the extended version of the parasite-replicase system implemented using multi-agent modelling approach (MAS). Similarly as the previous one [25], it can be classified as a MSL1 model (multi-level selection of type one), in which the entities are treated as focal units [54]. It also considers two-dimensional space and accounts for diffusion based on Brownian dynamics [25, 31, 55, 56]. The crucial difference between this study and the previous research [25] lies in the considering more realistic biological assumptions throughout the explicit introduction of the RNA sequence and structure. Each entity represented by an agent (parasite or replicase) is equipped with parameters which values are directly derived from its primary and secondary RNA structure. Additionally, in order to reflect as thoroughly as it is possible the initial prebiotic conditions in which first RNA molecules operated, we have taken into account the results obtained by Kierzek and co-workers concerning the process of RNA-structure driven spontaneous, non-enzymatic RNA degradation (expressed as the agent’s decay rate *b*) [37–40].

Moreover, since it was postulated that the presence of specific domains dedicated to the certain functions were probably required in case of RNA replicases adaptive evolution [50, 57, 58], we have introduced characteristic catalytic motif (presumed in arbitrary manner), which is directly associated with replicase efficiency (expressed as the agent’s parameter *a*). Thus, only the RNA molecules carrying such a motif can act as RNA polymerase ribozymes (replicases), similarly as in grid Monte-Carlo model with a resolution at the individual molecules level presented in [50]. Contrary to [50], where one mutation within functional domain causes an RNA replicase to become parasite, in our approach, several mutations inside the functional motif are allowed. The mutations together with primary and secondary structure of the catalytic motif influence the RNA replicase efficiency, which is common in modern life.

It is worth mentioning, that a similar approach was taken by Takeuchi and Hogeweg [36]. They introduced explicit RNA sequences into RP model and used secondary structure prediction to determine properties of each RNA strand. Computer simulation showed that their system could survive and produce several coexisting species. However, the goal of this research was slightly different. Our aim was to observe a possible evolution of replication efficiency over time within a large population. In order to be able to simulate millions of RNA strands, simpler model was used. Replicases had their inherent replication efficiency, thus some replicases could have better structure than others, which is the main difference as compared to Takeuchi and Hogeweg approach. Another one is, that our model accounts for more realistic diffusion.

It is worth noting that our objective was not to discover the exact motif having certain sequence content, length and function, but to observe the mechanisms of evolution and to show that such motif could have emerged in the early prebiotic enviroment and contribute to evolutionary change (see Scenario 2). Thus, on account of our ignorance in this aspect, we decided to use the arbitrarily presumed sequence to characterize replicases, but in general, any other sequence could be chosen.

In [50], apart from the characteristic domain, ribozyme is also equipped with a tag (3 nt sequence at the 3’-end of an RNA) and a reverse-tag (3 nt sequence at the 5’-end of an RNA). The entities containing a 3’-tag (“pseudo-parasites”) or both tags (replicases or “true parasites” being the molecules deprived of the characteristic domain) are the only ones that are recognized and can be replicated, while the tag-free species are neglected and become extinct. In tag-driven system, the space of the molecules that can be copied by RNA replicases is significantly reduced and in general, parasites have difficulty appearing de novo. The computer simulations conducted by the authors showed that the tag mechanism did not work by favoring ribozymes, but through weakening the parasites, what allowed RNA replicases to resist them and become prosperous. It was also postulated that tag mechanism has a synergic effect with the spatial limitation mechanism, where former provides space for the replicases to escape from parasites and latter gives them the time to accomplish it. It is the main reason why the authors claim, that the tag mechanism may constitute the solution to the parasite problem, which considers how could RNA replicases be selfish enough to favor their own replication, ignore other molecules and thrive in the system [12, 50, 59].

Unfortunately, in the model of [50], the RNA chain folding problem was not considered and it was assumed that the presence of the certain, arbitrarily assumed tag sequences is necessary to initiate the replication process and for the system to survive, which seems to be a great simplification. In contrast, within our approach, primary and secondary RNA structure is directly taken into account. Here, the replication process is indirectly limited through the requirement to form complexes, which depends on the values of agent’s parameters *a* (replicase efficiency) and *l* (fraction of the time spent by the RNA molecule in the folded state) being the stabilizing factor in the system. These assumptions are consistent with [25, 31–33]. Similarly as in [25], agents must be in the unfolded state to serve as templates, whereas in the folded state parasites are considered as inactive while RNA replicases can perform as catalysts. The above mentioned parameters, namely *a* and *l*, can change during the simulations as a result of the mutations in the RNA sequence.

The Scenarios 1-3 with evolving parasites and replicases show that the system is able to survive and allows for the stable coexistence for both quasispecies. The situation is similar, whether the simulation concerns the population of replicases only, where parasites appear due to the high mutation rate, or the population of the initial RNA sequences is completely random and some of them accidentally are equipped with the replicase functional motif. Since the RNA replicases are challenged by parasites and the ribozyme-derived parasites are unavoidable, in order to survive, they should tend to assemble together to promote the catalysis of their own replication. In our system, it is possible through the finite diffusion, which causes that replicases are located within close proximity of their relatives (agents with similar RNA sequences and parameters). Their limited dispersal allows for the preservation of strongly altruistic ribozymes, observe the kin selection and consequently, most likely overcome the parasite problem.

Additionally, Scenario 4 was carried out in order to show the parameters ranges where the system collapses versus survives. The results of the simulations clearly show that the values of two parameters, namely base decay rate *d* and diffusion constant *D* are crucial regarding the system survival and evolution. Base decay rate should have the optimal value, because if it is too low then the population is much smaller, but on the other hand if it is too high then the system goes extinct. Diffusion constant can seriously affect the system if it is close to 0, because it prevents the propagation of the replicases, so the population is unable to expand.

In [25], it was shown that even if spatially extended RP system is stable, evolution of better replicases is not guaranteed. This is a consequence of the dual role played by the replicase, which performs replication (serves as an enzyme) and stores the information necessary for creating new instances of itself (serves as a template). Their results indicated, that this strong trade-off favors the evolution of the ribozymes toward good templates (by maximizing the time spend in the unfolded state) rather than toward good catalysts (by minimizing the time spend in the folded state). It was also visible in their simulation results, where *l* parameter of replicases decreased quickly reaching very low level close to 0, while *a* parameter increased rapidly to the maximum level close to 1. In case of parasites, *l* parameter dropped slightly below 1. It means that, the replicases were definitely better recognized and favored by other replicases as templates, while parasites were rarely copied, which is also consistent with the results presented in [22, 60]. These extreme values of agent’s parameters were probably observed, due to the simplifications introduced in the model. RNA molecules properties were modeled by parameters, which values were assigned arbitrary, apart from the real sequence.

On the contrary, simulations conducted in this study show that it is possible to reconcile two conflicting goals at the same time, namely being a carrier of genetic information (good template) and catalyst (having a good replicase activity). It is noticeable that the average value of replication efficiency (*a*) drops in the simulations, but remains still high (over 0.8), while the probability of being in the folded state (*l*) is also reduced and stabilizes in case of both agents at a level below 0.3. It means that the selection favors better templates and good enzymes in the same time. Additionally, the *l* parameter was on average lower in case of parasites, which means that they evolved toward being good templates that are better recognized and easier copied than replicases.

It is worth noting that the model presented in this study considers simplified RNA replication mechanism, but does not impose any restriction on the molecules that can be copied by the replicases. Similarly as in [25], it does not distinguish between gene (+) and enzymatic (-) RNA strands, which can be raised if the exact form of the RNA replication is taken into account. However, the results obtained in this study are consistent with the results obtained in the approach taken by [61], where gene-enzyme specialization has been introduced and simulated inside compartments (so-called protocells). It has been shown there, that a physical separation of the informational strand and the enzyme strand can lead to the one being a good template and the other a good catalyst. However, this approach does not take into account the emergence of a parasitic genome replication strategy where no replicase is ever produced.

As already mentioned, in this work we have extended the MAS approach presented in [25] by introducing more realistic biological assumptions by taking into account the RNA sequence and structure. At present, the parameters assigned to every agent in the analyzed system are derived directly from its primary and secondary RNA structure. Hence, the simulations conducted in this research have allowed to follow in a more accurate and precise manner the simple evolution mechanism of early life. It is especially visible in the Scenario 2, which considers the set of completely random RNA sequences, where some of them can be identified as replicases (as they are accidentally having functional replicase motif). It shows that the occurrence of the replication mechanisms could arise spontaneously. Additionally, it is worth noting that the replication activity in the analyzed model is recognized through arbitrarily presumed motif, but in general it could be influenced by other factors.

## Conclusion

The results presented in this paper provided grounds for drawing the conclusion, that in a prebiotic environment some conditions could lead to the spontaneous occurrence of the replication mechanisms and the appearance of replicases containing simple functional domains. Simulations conducted by the authors in which all RNA sequences were completely random, show that if some replicases emerge from the random pool of RNA sequences, then their population can grow and take over the system, which can finally reach stability. Thus, it shows that evolutionary selection works even in the simplest systems. Parasite and replicase populations have the ability to regulate each other and their mutual interaction can result in equilibrium. In the beginning, the replicase population was very small, thus the system “encouraged” its growth. The real process of emerging natural polymerase ribozymes could have happened in a very similar way—some of them appeared randomly and quickly started to copy themselves in order for the system to reach equilibrium.

The obtained results confirm also that a stable coexistence of replicases and parasites is possible in a spatially extended system. Moreover, even if a population of replicases only is considered, the appearance of ribozyme-derived parasites is unavoidable. The results also show, that it is possible to be a good template and catalyst at the same time and also that parasites tend to be good templates, that are easier to be copied than replicases. Since, as shown in [25], a loss of a catalytic activity is definitely easier than the loss of an affinity towards replicases, the once formed parasites could be further copied and subsequent mutations could occur. Some mutations may have provided advantages to the system and, occasionally, new functions could be developed. Therefore, it could lead the parasites to evolve toward being storage information, namely genes. It is consistent with the results presented in [61], where the authors conclude that division of labor between genes and enzymes was under strong positive selection in the RNA world.

Moreover, exhaustive simulations were carried out in order to show the parameters ranges where the system collapses versus survives. The results show that two parameters namely base decay rate and diffusion constant are very important regarding system survival. The former should have the optimal value, because if it is too low then the population is much smaller, but on the other hand if it is too high then the system goes extinct. The latter can seriously affect the system if it is close to 0, because the replicases can’t propagate then, so the population is unable to expand.

As a continuation of the research reported in this paper, one may consider taking into account the exact RNA replication mechanism as described in [61]. Additionally, in order to reflect more accurately the RNA folding and what follows, the interactions between RNA molecules, one may consider extended secondary (taking into account both canonical and non-canonical base pairs contrary to 2D structure which considers canonical base pairs only) and tertiary interactions (formed between secondary structure elements and constituting RNA tertiary (3D) structure) within and between RNA chains [62–66]. However, conducting such simulations will certainly require very high computing power.

## Supporting information

S1 Appendix Parallelization details of the simulation algorithm. Here, parallelization details together with efficiency tests of the simulation algorithm are described.
(PDF)

S1 Fig Computation time depending on the number of threads and architecture. (EPS)

S2 Fig Parallelization efficiency depending on the number of threads and architecture. (EPS)
