# Supplementary material for: Multi-agent approach to sequence structure simulation in the RNA World hypothesis
Source: PLoS One. 2020 Aug 28;15(8):e0238253. doi: 10.1371/journal.pone.0238253 (PMC7455006; doi:10.1371/journal.pone.0238253)
Supplement: S1 Appendix — Here, parallelization details together with efficiency tests of the simulation algorithm are described. (PDF) [file pone.0238253.s001.pdf]

Supporting Information 1 for: Multi-agent approach to sequence structure simulation in the RNA World hypothesis

Jaroslaw Synak[1,3], Agnieszka Rybarczyk[1,2,3*], Jacek Blazewicz[1,2,3*]

1 Institute of Computing Science, Poznan University of Technology, Poznan, Poland
2 Institute of Bioorganic Chemistry, Polish Academy of Sciences, Poznan, Poland
3 European Center for Bioinformatics and Genomics, Poznan, Poland

* jblazewicz@cs.put.poznan.pl, arybarczyk@cs.put.poznan.pl

S1 Appendix

S1a: Parallelization

The simulation was paralellized using geometrical partition. The area was divided into identical rectangles and every thread computes its own set of them. The execution has to be synchronized after performing the diffusion and after every step is finished as shown in Algorithm 1.

S1b: Efficiency tests

During tests two machines were compared:

- IBM's Power AI
- Auriga - PUT's server with Intel Xeon E5-4640 processor

Computation time (S1 Fig 1) is not so important, because it strongly depends on the raw processor speed, more important parameter is computation efficiency (S1 Fig 2), which depends much more on the architecture itself. PowerAI has very clear advantage over Intel, especially when more threads are used (more than 7). Theoretically efficiency should not be greater than 100%, but in practice it can, because of the cache optimizations.

S1 Fig 1. Computation time depending on the number of threads and architecture.

S1 Fig 2. Parallelization efficiency depending on the number of threads and architecture.
